# Active ingredients of traditional Chinese medicine for enhancing the effect of tumor immunotherapy

**DOI:** 10.3389/fimmu.2023.1133050

**Published:** 2023-03-10

**Authors:** Chao Yang, Dan Li, Chung-Nga Ko, Kai Wang, Haiyong Wang

**Affiliations:** ^1^ National Engineering Research Center for Marine Aquaculture, Institute of Innovation & Application, Zhejiang Ocean University, Zhoushan, Zhejiang, China; ^2^ State Key Laboratory of Southwestern Chinese Medicine Resources, School of Pharmacy, Chengdu University of Traditional Chinese Medicine, Chengdu, China; ^3^ C-MER Dennis Lam and Partners Eye Center, Hong Kong International Eye Care Group, Hong Kong, China; ^4^ Research Center for Preclinical Medicine, Southwest Medical University, Luzhou, China; ^5^ Department of Internal Medicine Oncology, Shandong Cancer Hospital and Institute, Shandong First Medical University and Shandong Academy of Medical Sciences, Jinan, China

**Keywords:** tumor immunity, immunomodulation, traditional Chinese medicine, active ingredients, antitumor immunotherapy, natural products

## Abstract

Immunotherapy is a type of treatment that uses our own immune system to fight cancer. Studies have shown that traditional Chinese medicine (TCM) has antitumor activity and can enhance host immunity. This article briefly describes the immunomodulatory and escape mechanisms in tumors, as well as highlights and summarizes the antitumor immunomodulatory activities of some representative active ingredients of TCM. Finally, this article puts forward some opinions on the future research and clinical application of TCM, aiming to promote the clinical applications of TCM in tumor immunotherapy and to provide new ideas for the research of tumor immunotherapy using TCM.

## 1 Introduction

Malignant tumors are cancerous tumors that have cells growing uncontrollably. If the condition remains untreated, malignant cells can spread to distant sites *via* the lymphatic system and bloodstream and become life-threatening (1). The rate of new cases of malignant tumors has been increasing over the past years, and the incidence of early-onset cancers is on the rise worldwide (2, 3). Traditional tumor treatment methods, such as radiotherapy, chemotherapy and surgery, have drawbacks of easy recurrence, large side effects and low survival rate (4). Immune homeostasis is regulated and maintained by the immune system, which is made up of immune organs, immune tissues, immune cells and immune factors. On the other hand, immune disorders can lead to the occurrence and progression of different immune diseases (5–7). Tumor immunotherapy, which specifically kills and removes tumor cells by activating the autoimmune system and enhancing autoimmunity, is considered the only possible method to completely remove tumor cells (8, 9). Therefore, tumor treatment strategies that regulate immune activity have attracted increasing attention from researchers.

Traditional Chinese medicines (TCM) are known as a renowned source of medicinal compounds. They offer advantages of low cost, structural and functional diversity, and few side effects, and has a critical role in the long history of Chinese civilization (10, 11). However, the complex components of TCM, the unclear pharmacological mechanism of action, and the slow onset of action have significantly hindered its development (12, 13). With advances in science and technology and the improvement of the public’s understanding of TCM, there has been a worldwide upsurge in the research and development of TCM. Several studies have shown that some TCM are beneficial in improving the clinical symptoms of COVID-19 (14–16).

Currently, a variety of TCMs have been reported to have antitumor activity as well as to enhance immunity and survival rate of patients. Some studies suggest that TCM offer advantages over Western medicine at a certain stage of cancer treatment (17–19). Therefore, identifying the active substances in TCM and understanding their antitumor pharmacological mechanisms are crucial for the development of TCM in the future. This article describes the main regulatory mechanisms of tumor and body immunity, summarizes the representative active ingredients of TCM that play an immunoregulatory role in tumor treatment, and discusses their pharmacological mechanisms.

## 2 Immune regulation in tumors

Cancer immunoediting is the process of interaction and mutual influence between tumor cells and the body’s immune system (20, 21). Cancer immune regulation consists of three stages, namely, 1) elimination phase, 2) equilibrium phase and 3) escape phase (22, 23). During the elimination phase, the mutated “non-self” cells in the body are recognized and eliminated specifically by the surveillance function of the immune system (24). However, tumor cells with more mutations can alter their own characteristics and evade immune surveillance (25). As a result, the immune system cannot completely clear tumor cells. In this stage, tumor cells cannot significantly proliferate and expand due to the immune-mediated killing and immune stress (26, 27). The inhibitory activity of the immune system and the proliferative activity of tumor cells have reached a dynamic balance (28, 29). Subsequently, due to immunosuppression, exhaustion, or tumor cells mutation, the balance between the immune response and tumor activity is disrupted, allowing the mutated tumor cells to escape the immune pressure (30). During the escape phase, these tumor cells continue to clonally proliferate, eventually forming a clinically detectable, progressively growing tumor (31). In addition, tumor growth also establishes an immunosuppressive microenvironment, which further aids tumor cell escape and proliferation (32, 33).

### 2.1 Mechanisms of tumor immune escape

Accumulated evidence has shown that the immune escape mechanisms of tumors mainly include the following aspects:

#### 2.1.1 Lower immunogenicity

Tumor cells with strong immunogenicity can induce antitumor immune responses and are easily eliminated (34). On the other hand, tumor cells with weak immunogenicity can escape the immune recognition and surveillance and achieve selective proliferation (35, 36). Therefore, reducing or losing the expression of MHC class I molecules on the surface of tumor cells is one of the main reasons for their immune escape (37, 38). Losing or downregulation of MHC class I molecules could result in a failure to present intracellular antigens on cell surface, and thus a failure to activate T cells (39, 40). Consequently, immune tolerance is achieved due to the non-response of T cell immunity, giving tumor cells the ability to evade surveillance of the immune system.

#### 2.1.2 Tumor cell antigen modulation or antigen deletion

Some tumor cells express antigens similar to those of normal cells, which cannot induce the body to generate an effective antitumor immune response (41, 42). Furthermore, when the immune system recognizes and attacks the antigens that are originally expressed by tumor cells, some tumor cells can reduce or even eliminate these antigens through antibody-induced antigen internalization and mutation of the antigen itself, thereby escaping the fate of immune killing (43).

#### 2.1.3 Abnormal tumor cell costimulatory signaling

In addition to TCR recognition, costimulatory signals expressed by tumor cells could also cause T cells activation (44). For example, the B7 family members are important costimulatory molecules responsible for regulating T cell proliferation and cytokine production (45). Many tumor cells lack B7 or other costimulatory signaling molecules and cannot activate T cells (46). Moreover, while positive costimulatory molecules (e.g. CD80 and CD86) are rarely expressed by tumor cells, negative costimulatory signals (e.g. PD-L1) are expressed. As a result, antitumor immune responses cannot be effectively induced (47).

#### 2.1.4 Tumor-induced exemption regions

A variety of molecules, for example collagen, can be excreted by tumor cells and serve as a physical barrier around the tumor in order to prevent the entrance of antigen-presenting cells (APC) and lymphocytes into the tumor area (48).

#### 2.1.5 Suppression of immune response

Tumor cells actively induce the body to produce tumor-associated macrophages, myeloid suppressor cells and regulatory T cells (Tregs) to suppress the body’s immune response (49).

#### 2.1.6 Targeting T cells

Protein factors secreted by some tumor cells, such as PD-L1 and FasL can inhibit T cell proliferation or even induce T cell apoptosis (50, 51).

#### 2.1.7 Tumor cell-induced immunosuppression

Immunosuppressive molecules produced by tumor cells, such as IL-10, TGF-β, IDO or PD-L1, can directly suppress the immune response (52, 53). Meanwhile, tumor cells can also recruit regulatory T cells that secrete immunosuppressive cytokines (54, 55).

### 2.2 Tumor microenvironment

Tumor microenvironment (TME) is the living environment of tumor cells, mainly including tumor cells and various surrounding cells, such as B lymphocytes, T lymphocytes, natural killer cells (NK cells), tumor-associated macrophages (TAMs), myeloid-derived suppressor cells (MDSCs), tumor-associated neutrophils (TANs), and dendritic cells (DCs)); blood and lymphatic vessels; as well as the intercellular substance, microvessels, and bioactive molecules infiltrating into the surrounding area (56). Hypoxia, acidification, interstitial hypertension, vascular hyperpermeability, inflammatory reactivity, and immunosuppression are the main features of TME (57). TME contains a variety of immunosuppressive molecules, which can protect tumor cells from cytotoxic T lymphocytes as well as enhance the immunosuppressive function of Tregs and MDSCs (58). This in turn promotes tumor growth and development.

### 2.3 Immune cells in the TME

#### 2.3.1 Regulatory T cells

Tregs are a class of mature T cell subsets with immunosuppressive activity. They regulate the body’s immune system by actively regulating the proliferation and activation of autoreactive T cells. Treg cells are one of the important factors in maintaining the immune homeostasis of the body. When the immunosuppressive function of Treg cells is weakened or declined, the functions of killer T cells and helper T cells (Th cells) are enhanced, which can cause autoimmune diseases, such as systemic lupus erythematosus, multiple sclerosis, and rheumatoid arthritis (59). However, when the immunosuppressive function of Treg cells is too strong, the functions of killer T cells and Th cells are inhibited so that the immune system cannot effectively phagocytose pathogens, thereby causing diseases, such as viral infection and tumor escape (60).

#### 2.3.2 Helper T cells

Th cells are a major component of the immune system, and their main surface marker is CD4 (61). Antigen receptors on Th cells surface can recognize fragments of antigens presented by MHC class II molecules of APC (62). They can also assist CD8+ T cells and B cells in producing cytokines, thereby activating or regulating stromal cells, epithelial cells and innate immune cells, among others (63). Activated Th cells regulate or assist immune responses by secreting cytokines (64).

#### 2.3.3 Cytotoxic T cells

Cytotoxic T cells (CTL or Tc cells), also known as killer T cells, are surveillance cells that can directly attack tumor cells with heteroantigens, virus-infected cells and foreign cells (65). Tc cells can induce lysis or apoptosis of target cells by secreting perforin or granzyme (66). Memory T cells are antigen-experienced cells differentiated from Tc cells that have memory for antigen-bearing target cells. Once they encounter target cells with specific invading antigens, they stimulate Tc cells to produce effector T cells to destroy infected cells or cancer cells (67).

#### 2.3.4 B cells

B cells is a type of professional antigen-presenting cell derived from pluripotent stem cells of the bone marrow (68). Activated B cells bind to soluble antigens through their surface BCRs and present them to CD4+ T cells in the form of antigenic peptide-MHC molecule complexes (69). Activated B cells can generate a large number of cytokines and play a role in inflammatory response, immune regulation, and hematopoiesis (70). B cells infiltrating tumors can exert antitumor immunity by driving complement activation, phagocytosis and antibody-dependent cell-mediated cytotoxicity (71).

#### 2.3.5 Macrophages

Macrophages are a type of white blood cell derived from bone marrow stem cells. They develop into monocytes and then distribute to various organs and tissues *via* the blood. Macrophages play an important role in engulfing and digesting pathogens and maintaining tissue homeostasis (72). TAMs infiltrating tumor tissues are highly plastic and heterogeneous. Proinflammatory cytokines, such as Toll-like receptor (TLR) agonists, can promote TAMs polarization to the M1 type, while colony-stimulating factor 1 and interleukin-4 (IL-4) induce polarization of TAMs toward the M2 type (73). The proinflammatory factors tumor necrosis factor (TNF-α), IL-6, IL-10, IL-23, nitric oxide (NO) and reactive oxygen species (ROS) secreted by M1 macrophages can significantly increase the inhibition of tumor cell proliferation. M2 macrophages secrete epidermal growth factor (EGF), matrix metalloprotein 9 (MMP-9) and the anti-inflammatory factor IL-10 to promote tumor progression (74).

#### 2.3.6 Neutrophils

Neutrophils, the most abundant type of granulocyte that make up 40% to 70% of all white blood cells in humans, play a critical role in the innate immune system (75). Neutrophils have a variety of specific receptors (e.g. complement receptors), and can produce cytokines (e.g. IFN-γ and interleukins), chemokines, lectins, and other proteins (76). In addition, neutrophils express receptors for detection and adhesion of the endothelium and Fc receptors for opsonins (77). TNF-α and interferon-β induce neutrophils to polarize toward the antitumor N1 type with high immune activity and promote CD8+ T cell activation (78). IL-8 and TGF-β also promote the polarization of TANs to the tumor-promoting N2 type, thereby inhibiting tumor immunity and accelerating tumor progression (79).

#### 2.3.7 Dendritic cells

CD34+ hematopoietic stem cell DCs derived from bone marrow are the most functional professional APC in the body, which can efficiently take up, process and present antigens, including surface-expressing antigen-presenting molecules (MHC class I, MHC class II and CD1 molecules), costimulatory molecules (B7-1, B7-2) and CD40 (80). In addition, DCs are the only APCs that can activate naïve T cells and play a key role in T cell activation and differentiation (81). Meanwhile, the growth factors and cytokines produced by DCs can enhance the activity of immune cells, such as NK cells and T cells, and establish a complete antitumor immune response (82).

#### 2.3.8 Myeloid-derived suppressor cells

MDSCs, an immunosuppressive innate cell population composed of immature myeloid progenitors, is a class of cells that could not differentiate into macrophages, granulocytes and DCs (83). MDSCs can display potent immunosuppressive activity through multiple regulatory mechanisms, such as producing reactive oxygen species and nitrogen, inducing immunosuppressive cells, depleting metabolites critical for T cell function, blocking lymphocyte homing, expressing regulatory glands, expressing extracellular enzymes of glycoside metabolism, and expressing negative immune checkpoint molecules (84). Therefore, MDSCs are implicated in malignant tumors and are potential targets for tumor therapy.

#### 2.3.9 Natural killer cells

NK cells are primarily involved in the body’s first line of defense against tumors. The molecular basis for NK cells to identify and eliminate transformed cells are based on the expression of MHC-I-specific inhibitory receptors on the NK cell surface. Tumor cells having down-modulated expression of surface MHC-I become susceptible to attack by NK cells (85). Furthermore, Fc receptors on the surface of NK cells can recognize the Fc portion of IgG antibodies and provide NK cells the ability to recognize and kill the IgG-coated tumor cells. NK cells also have natural cytotoxicity receptors (NCRs) that bind to antigens on the surface of tumor cells, thereby lysing tumor cells (86). NK cells and B cells can non-specifically kill tumor cells without antigen presentation (87). In addition, NK cells can help the response and formation of tumor-specific CD4+ and CD8+ T cells (88).

#### 2.3.10 Mast cells

Mast cells, also known as mastocyte, are resident cell of connective tissue derived from CD34+ myeloid precursor cells. They circulate in blood and migrate to the vicinity of systemic lymphatics, blood vessels, and mucosal surfaces (such as the gastrointestinal tract and skin) and are involved in the coordination of innate and adaptive immunity (89). Mast cells have been found to have either protumor or antitumor effects, depending on the tumor type, cancer stage, mast cell activation status, the balance of tumor-promoting and antitumor effects on tumor cells, and the location of mast cells in the TME (90). Chemokines (e.g., CCL3 and CXCL8), cytokines, and other factors released by mast cells are able to recruit other immune cells (e.g. macrophages, MDSCs, neutrophils, NK cells, and DCs) into the TME and alter its activity or function (91).

## 3 Antitumor immunomodulatory activity of active ingredients of TCM

TCM is the crystallization of thousands of years of medical practice (92). The composition of TCM is complex. Although their active ingredients and pharmacological mechanism of action are unknown, clinical trials have shown that TCM can significantly prolong the postoperative survival time of tumor patients in the treatment and prevention of cancer recurrence and metastasis. At present, many studies have analyzed and verified the antitumor immunomodulatory activity of TCM monomers or single components, including polysaccharides, alkaloids, flavonoids, and terpenes (93–95). Due to the diversity of active ingredients in TCM and their promising pharmacological effects, representative compounds of TCM and their antitumor immunomodulatory activities are discussed in this section.

### 3.1 Alkaloids

Alkaloids are a class of natural nitrogen-containing alkaline compounds that possess a variety of physiological effects (96). Especially, alkaloids that contain complex nitrogen heterocyclic ring structure usually have significant biological activity and are important active ingredients in TCM. Alkaloid components in TCM are high-efficiency and low-toxicity antitumor compounds and are widely used in the medical field.

**Figure 1:**
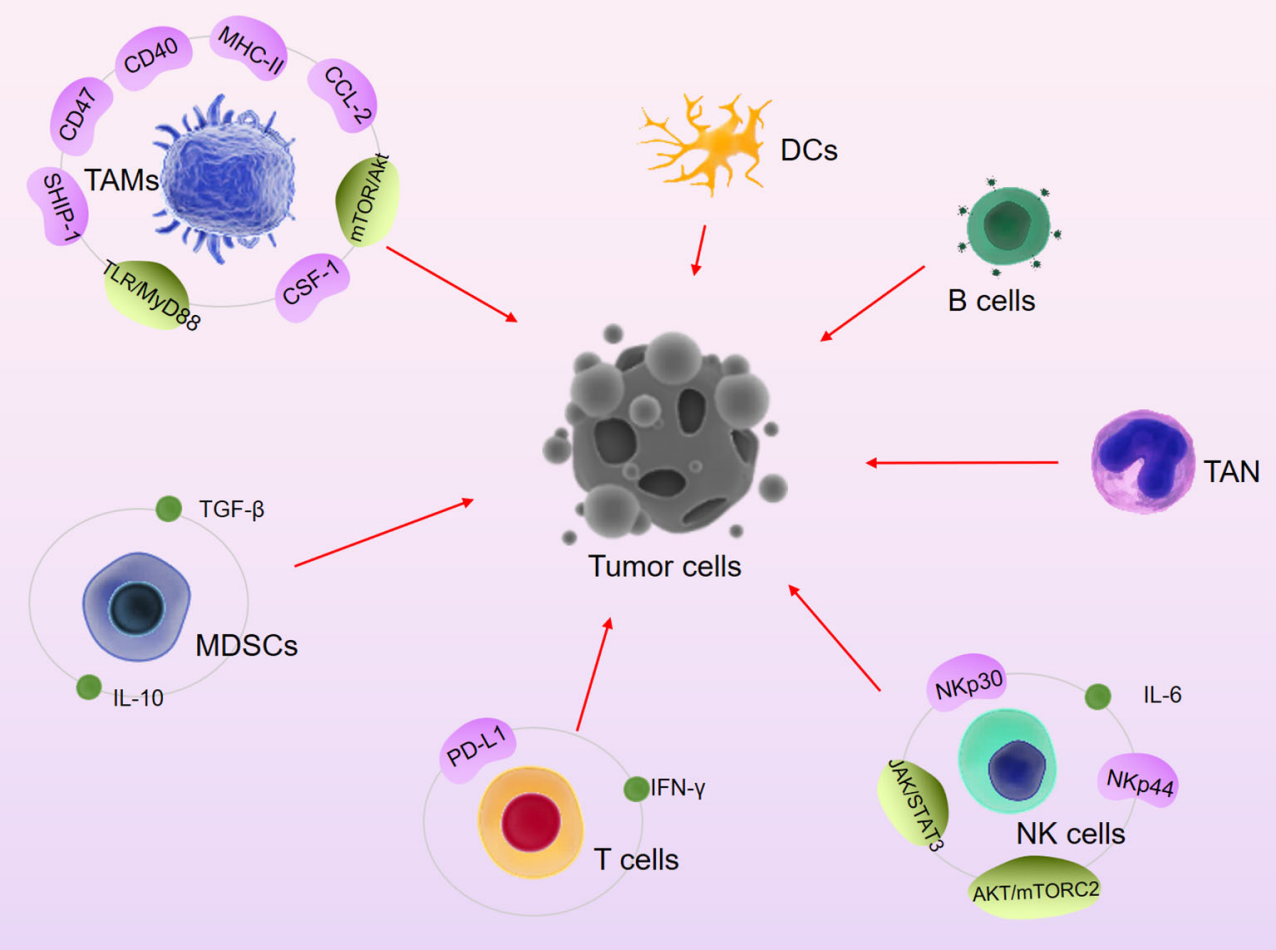
Major signaling pathways and immune cells involved in TCM-mediated anti-tumor immune activity.

Berberine (BBR) is an alkaloid isolated from the TCM Coptis chinensis with significant antitumor activity (11, 97). Continuous DOX treatment can cause HL-60 cells to differentiate toward N2, resulting in chemoresistance. BBR can downregulate DOX-mediated expression of CD133 and CD309 in neutrophils, thereby inhibiting PD-1/PD-L1-mediated chemotherapy tolerance and immune rejection (98). Furthermore, BBR triggers PD-L1 ubiquitination and degradation through a ubiquitin (Ub)/proteasome-dependent pathway (97). Recent studies suggest that BBR also inhibits the expression of CD47, whose mediates immune escape, results in limited efficacy of rituximab and enhances phagocytosis by macrophages (99). In a melanoma model, BBR increased CD40 and MHC-II expression on macrophages, increased the number of IFNγ-producing CD4 T cells and the CTL activity. Previous studies also suggest that BBR can promote NK cell infiltration, increase the expression of inflammatory factors such as IL-6, and activate the apoptosis pathway (100). Therefore, the antitumor immunomodulatory activities of BBR mainly include targeting immune checkpoints, increasing NK cell activity, regulating the neutrophil phenotype, inducing macrophage polarization, and inhibiting the expression of inflammatory factors (**Figure 1**).

Matrine is an alkaloid that possess various pharmacological activities and can be extracted from Sophora flavescens by organic solvents, such as ethanol. The antitumor activity of matrine includes inducing tumor cell differentiation and apoptosis, inhibiting tumor cell proliferation and metastasis, inhibiting telomerase activity, inhibiting tumor angiogenesis and inhibiting tumor drug resistance, as well as increasing the activity of white blood cells to enhance the body’s immunity.

Metastasis is responsible for nearly 90% of lung cancer deaths. Matrine inhibits lung cancer metastasis by enhancing T cell proliferation and inhibiting M2-like tumor-associated macrophage polarization (101). In leukemia treatment, matrine enhances the killing activity of NK cells on K562 cells by inhibiting IL-6-mediated JAK/STAT3 signaling in K562 cells. On the other hand, matrine significantly enhances antitumor immune activity by enhancing DC cell maturation, activation and differentiation (102). In addition, matrine promotes the secretion of inflammatory cytokines by regulating the TLR signaling pathway, which further enhances the body’s immune function (103).

### 3.2 Flavonoids

Flavonoids, a class of yellow pigments derived from flavonoids (2-phenylchromone), are present in a variety of fruits, vegetables, TCMs and other plants. Flavonoids possess a wide variety of biological activities, including anti-inflammatory, antioxidant, antitumor and hypoglycemic activities, as well as alleviation of vascular hyperplasia.

Epigallocatechin gallate (EGCG) is the most effective active ingredient in tea polyphenols and has been found to possess antioxidant, antiviral, antibacterial, antiarteriosclerosis, antithrombotic, antivascular proliferation, anti-inflammatory and antitumor effects. EGCG is an immune checkpoint inhibitor that can significantly downregulate the expression of PD-L1 induced by epidermal growth factor (EGF) or IFN-γ (104). Meanwhile, EGCG inhibits the JAK/STAT signaling pathway in tumor cells, activates T cells, and enhances the antitumor immune response (105). In an oral cancer study, it was found that EGCG significantly inhibited the expression of IDO by blockage of the IFN-γ-induced JAK-STAT1 signaling pathway (106). Therefore, EGCG can inhibit IDO-mediated immune escape to a certain extent or can be used as a potential targeted immunotherapy drug. In leukemia models, different doses of EGCG have different immunomodulatory activities, such as promoting natural killer cell activity, promoting phagocytosis of macrophages, and T cell proliferation (107). Furthermore, EGCG can inhibit M2 polarization and TAMs infiltration by inhibiting the expression of monocyte chemokines (CSF-1 and CCL-2) and HIF-1α, thereby inhibiting the development of breast cancer in mice (108, 109).

Apigenin, also known as chamomile, apigenol, apigenine, versulin, and others, can be found in a variety of vegetables, fruits, and plants. It has been confirmed that apigenin has biological activities, such as vasodilation; lowering blood pressure; lowering blood lipids; and acting as an anti-inflammatory, antitumor, and antioxidant factor.

After coculturing liver cancer patients-derived NK cells with apigenin, the expression levels of its activating receptors NKp44, NKp30 and NKG2D were significantly increased, while the level of TGF-β1 was decreased (110). Mechanistic studies have shown that apigenin increases the sensitivity of HCC cells to NK cells through HIF-1α. Apigenin can strongly inhibit STAT1 activation and IFN-γ-induced PD-L1 expression in tumor cells and enhance T cell-mediated killing (111, 112). In addition, apigenin inhibits the infiltration of TAMs and other leukocyte subsets in tumors, such as IL-1α, IL-6, granulocyte macrophage colony stimulating factor (GMCSF) and CCL2 (113–115). The expansion of immunosuppressive MDSCs and TAMs in the inflammatory TME results in ineffective immunotherapy for many tumors. Apigenin can enhance the proportion of tumor-killing macrophages by inducing the expression of SHIP-1, thereby enhancing the antitumor immune response (116, 117). Moreover, apigenin is also involved in maintaining T cell homeostasis in mouse pancreatic cancer (118). Other antitumor immune activities of apigenin have been reviewed by Huang et al. and are not discussed here (119).

### 3.3 Saponins

Saponins are naturally-ocurring glycosides with complex structures. They are widely distributed in all cells of legume plants. As an active ingredient in many TCMs, actions of saponins include anti-inflammation, inhibition of the growth of various tumor cells and improvement of the activity of immune cells.

Ginsenosides is a class of steroid glycoside present in ginseng. Ginsenoside has pharmacological activities, such as improving immunity, anti-aging, antibacterial, anti-fatigue, and antitumor properties. Ginsenosides can directly activate CD4+ T cells, promote T cells differentiation into Th cells, and enhance the immune response [30]. When tumor cells are killed by external stimuli, the process that the cells transform from a non-immunogenic to immunogenic state to mediate the body’s antitumor immune response is called immunogenic cell death (ICD). Ginsenosides can kill both immunogenic and nonimmunogenic tumor cells by enhancing DC function and inducing apoptosis (120). A recent study revealed that ginsenosides could attenuate the expression of cisplatin-inducing PD-L1 in non-small cell lung cancer cells (A549/DDP cells) and enhance the killing activity of T cells against tumor cells (121). When used in combination with other chemotherapeutic drugs, ginsenosides can enhance antitumor activity and reduce side effects. For example, cyclophosphamide (CTX) has unstable efficacy and serious side effects, and its application has been greatly limited. Wang et al. showed that ginsenosides could promote the antitumor activity of CTX by enhanacing the proliferation of intestinal probiotics and also alleviate CTX-induced intestinal mucositis by activating the Nrf2 signaling pathway (122). Furthermore, ginsenosides enhance macrophage innate immune responses by activating the LPS-induced mTOR/Akt signaling axis (123).

### 3.4 Terpenoids

Terpenoids are the most abundant class of compounds in natural substances and are the main components of the essences, resins, and pigments of certain plants. In addition, some animal hormones and vitamins also belong to terpenoids. Therefore, terpenoids are important compounds in TCM and are indispensable raw materials for the chemical and food industries.

Triptolide (TP) is an epoxy diterpene lactone compound that can be isolated from the roots, flowers, leaves and fruits of Tripterygium wilfordii. Tp is also a natural product with various pharmacological activities, such as anti-senile dementia, antioxidant, antitumor and antibacterial activities.

Recently, Jiang et al. discovered the mechanism by which TP remodels the TME. TP remodels the TME in the colorectum by downregulating the expression of CD206 and IL-10 and inducing macrophage polarization (124). Similar to other active ingredients of TCM, TP can inhibit the expression of PD-L1 induced by IFN-γ or chemotherapy resistance, significantly enhance the secretion of TNF-α and IL-2, and improve the NK cells activity (125–127). Chen et al. found that TP exerts tumor immunosuppressive effects by inhibiting DC maturation and trafficking (128). In addition, TP can enhance antitumor immunity by inhibiting the expression of Treg cells and IL-10 and TGFβ (129). TP was also found to significantly upregulate the cell populations of B cells (CD19), T cells (CD3), macrophages (Mac-3) and monocytes (CD11b) in leukemia cells, enhancing the phagocytosis of macrophages (130).

### 3.5 Phenols

Phenolic compounds are widely found in foods in the human diet as well as in plants, and their applications in human health has been widely studied.

Resveratrol, a phytoalexin produced by plants to resist external stress, is widely found in berries, peanuts, mulberries, grapes, and other fruits.

Resveratrol has been found to enhance antitumor immune activity by inhibiting PD-1 expression or blocking the PD-1 signaling pathway in various tumors (131, 132). Meanwhile, downregulated PD-1 may amplify the Th1 immune response and promote the activity of CD8+ T cells (133). Resveratrol treatment can enhance/restore the killing activity of NK cells in human and mouse whole blood and significantly inhibit growth and metastasis of tumor (134). It was suggested that resveratrol upregulates the expression of c-Myb by activation of the AKT/mTORC2 signaling pathway, thereby activating NK cells (135). In addition, resveratrol can enhance the susceptibility of breast cancer cells to NK cells through inhibiting the expression of c-Myc (136). Furthermore, resveratrol can reduce the number of CD8+CD122+ T cells and M2 TAMs by inhibiting the secretion of cytokines (IL-10 and TGF-β1) and the phosphorylation of STAT3 while inhibiting M2 macrophage polarization (137–139).

### 3.6 Polysaccharides

Polysaccharides are the most abundant naturally occurring macromolecular compounds, which are widely present in higher plants, algae, fungi and animals. Polysaccharides have various pharmacological activities and are widely used as clinical drugs and nutritional health care products.

Astragalus polysaccharide (APS), a type of water-soluble polysaccharide, is one of the most important natural active ingredients derived from Astragalus. In recent years, APS has attracted much attention due to their promising activities in anti-aging, regulating blood sugar levels, and immune activity.

Currently, APS is used as a synergistic immune enhancer in breast cancer treatment. Further mechanistic studies have shown that APS regulates immunity by activating multiple signaling pathways, including the activation of macrophages and the TLR4-mediated MyD88 signaling pathway (140, 141). APS can be used as an immune adjuvant in tumor chemotherapy because it can enhance lymphocyte proliferation and macrophage phagocytosis and, when combined with 5-FU, it reduces the immunosuppressive activity of 5-FU (142). Huang et al. shows that APS inhibits cytokines (IL-4, IL-6, IL-13, IL-17, IL-1β, IFN-γ and GM-CSF), and immunosuppressive agent (IL-10 and IL-12) expression, which helps in improving the quality of life of patients with advanced cancer (143). In APS-treated melanoma mice, it was found that the number of MDSCs and the expression of related factors (TGF-β, IL-10 and Arg-1) were significantly downregulated, and the activities of CD8+ T cells were significantly upregulated (144). γδT cell, which is the main subgroup of intraepithelial lymphocytes, has a critical role in maintaining intestinal mucosal homeostasis and immune regulation. APS also involves in the promotion of γδT cells proliferation and activation of γδT cells *in vivo* (145). Furthermore, APS can enhance the antitumor immune activity of T cells by regulating M2 macrophage polarization and promoting DC maturation (146).

## 4 Conclusion and Outlook

In recent years, the significance of the body’s immunomodulatory activities in tumor treatment has been evidently supported, thus promoting the advent of a variety of promising new tumor immunotherapies. While TCM are rich sources of medicinal compounds and have good biological activities, no modulators for clinical use in tumor immunotherapy have been approved. This article summarizes the immunomodulatory activity and mechanism of action of various active components of TCM in tumor therapy. The mechanisms of TCM active components in tumor immune regulation, including the promotion of T cell proliferation, enhancement of the activity of T cells and NK cells, inhibition of immune checkpoints, enhancement of the activity of tumor-associated macrophages, regulation of the polarization of tumor-associated macrophages, promotion of DC maturation, and inhibition of MDSC, are complex and diverse (**Table 1**).

**Table 1:** Mechanism of action of TCM active components in regulating tumor immunity.

	Alkaloids		Flavonoids		Saponins	Terpenoids	Phenols	Polysaccharides
	Berberine	Matrine	Apigenin	Epigallocatechin gallate	Ginsenosides	Triptolide	Resveratrol	Astragalus polysaccharide
promoting T cell proliferation	✓	✓	✓	○	✓	○	✓	✓
enhancing the activity of T cells and NK cells	✓	✓	✓	✓	✓	✓	✓	○
inhibiting immune checkpoints	✓	○	✓	✓	✓	✓	✓	✓
enhancing the activity of TAMs	✓	○	✓	✓	✓	○	✓	✓
Promoting DC maturation	○	✓	○	○	✓	✓	○	✓
regulating the polarization of TAMs	✓	✓	✓	✓	✓	✓	✓	✓
inhibiting MSDC	✓	○	✓	✓	✓	✓	○	✓

✓ means reported.

○ means not reported.

In addition to the active compounds listed in this paper, more immunomodulatory activities of the active components of TCM have also been reported (17, 147–152). However, the targets of these active compounds and their immunomodulatory activities remain unclear. On the other hand, the active ingredients of TCM that have been discovered nowadays only represent a small portion of TCM, and more research is needed to explore other active ingredients and new action targets/mechanisdms of TCM. The constantly updated cutting-edge technologies, such as spatial transcriptome, single-cell sequencing, and nano-delivery technology, have also paved the way for the exploration of new targets for immune regulation and the clinical application of TCM (153–156).

## Author contributions

CY and KW conceived and designed the whole project. CY and DL drafted the manuscript. C-NK and HW revised the manuscript. All authors contributed to the article and approved the submitted version.
